# Responsible companion animal guardianship is associated with canine visceral leishmaniasis: an analytical cross-sectional survey in an urban area of southeastern Brazil

**DOI:** 10.1186/s12917-022-03238-z

**Published:** 2022-04-11

**Authors:** Paulo Henrique Araújo Soares, Eduardo Sérgio da Silva, Klauber Menezes Penaforte, Renata Aparecida Nascimento Ribeiro, Marcella Oliveira Gama de Melo, Diogo Tavares Cardoso, Ingrid Morselli Santos, Raissa Cotta Machado, Clara Lemos Carneiro Trindade, Anna Karolyna Rodrigues Cunha, Rafael Gonçalves Teixeira-Neto, Saulo Nascimento de Melo, Vanessa Vilela de Aquino, Vinícius Silva Belo

**Affiliations:** 1 Universidade Federal de São João del Rei (UFSJ), Campus Centro-Oeste Dona Lindu, Avenida Sebastião Gonçalves Coelho 400, Chanadour, Divinópolis, MG Brazil; 2 Prefeitura Municipal de Divinópolis, Divinópolis, MG Brazil; 3 Universidade Federal de Minas Gerais, Belo Horizonte, MG Brazil

**Keywords:** Animal welfare, Dual Path Platform test, Enzyme-linked immunosorbent assay, *Leishmania infantum*, Surveillance, Control, Zoonosis

## Abstract

**Background:**

Responsible companion animal guardianship (RCAG) comprises a set of concepts involving activities, behavior and care that guardians must provide to ensure the welfare of their animals. When such principles are disregarded, the risk of animals developing zoonotic diseases, such as canine visceral leishmaniasis (CVL), increases. This disease is a public health problem in many urban settings in Brazil because dogs are the main reservoirs of *Leishmania* and are involved in the transmission of the parasites to humans. Our analytical cross-sectional epidemiological survey aimed to investigate the prevalence of CVL in a city in southeastern Brazil and to establish the association between the disease and a number of predictor variables including dog traits, socioeconomic status of guardians, ecological features of the domicile and RCAG.

**Results:**

Our study showed that the global prevalence of CVL in the sample canine population was 6.7% (47/704). All variables related to better dog care were associated with lower chances of infection. Multiple regression analysis revealed that the chances of animals being seropositive for CVL were significantly (*p* < 0.05) higher when guardians had no formal education or possessed a university degree (*vs.* those with complete primary or secondary schooling) and when dogs were sheltered outside the house and had free access to the streets. An additional novel finding was that dogs that were acquired as puppies presented half of the chance of developing the disease in comparison with those acquired at the adult stage. Geographically weighted logistic regression coefficients showed that the strengths of the predictor/CVL associations varied depending on the studied geographical space. Both models demonstrated that the associations were always in the same directions.

**Conclusions:**

Our findings indicate that regardless of age and mode of acquisition, adult dogs should be submitted to clinical evaluation and tests for CVL. RCAG can exert positive effects on the control of CVL.

**Supplementary Information:**

The online version contains supplementary material available at 10.1186/s12917-022-03238-z.

## Background

Responsible companion animal guardianship (RCAG) encompasses a set of concepts involving activities, behavior and care that guardians must provide to companion animals to ensure their quality of life and health [1]. When such principles are disregarded, companion animals face many dangers from, for example, exposure to weather, abuse, bite wounds from other animals, traffic accidents and diseases, and cause problems to humans by environmental pollution and bite injuries [2]. In addition, failure to adopt RCAG principles has been associated with the dissemination and increased incidence of zoonotic diseases [3].

In Brazil, canine visceral leishmaniasis (CVL) is a public health problem in urban settings, where dogs are the main reservoirs of *Leishmania* parasites, since an increase in the prevalence of CVL is generally associated with a higher incidence of human VL [4, 5]. Understanding the risk factors associated with CVL is essential for the control and prevention of the disease [6] but, despite the availability of consistent data regarding some aspects of canine infection, other issues are still controversial or poorly understood [7]. Thus, while some studies have investigated variables regarding attitudes and the care of dogs, literature concerning the relationship between variables relating to RCAG and the occurrence of CVL is sparse.

In the light of the above, this study aimed to investigate the prevalence of CVL in Divinópolis, a city in southeastern Brazil where the disease is endemic, and to investigate the association between CVL and variables including dog traits, socioeconomic status of the guardian, ecological features of the domicile and adoption of RCAG principles.

## Results

A total of 1488 dogs in seven strata of Divinópolis were screened for CVL by technical staff of the local Secretaria Municipal de Saúde (SEMUSA) between January and September 2018. In the period September 2018 to May 2019, members of the Universidade Federal de São João del Rei (UFSJ) research team were able to revisit all of the selected domiciles with the aim of applying a questionnaire about CVL predictor variables. The number of participating dogs at the end of the survey was 704, representing 47.31% of the tested canine population. Sample losses were due to absence of residents in the domicile at the time of the survey visit (51%), unidentifiable or incomplete addresses in the records of SEMUSA (35%), change of address of guardians in the interval between screening and survey visits (8%), and refusal of residents to answer the questionnaire (6%). Sample losses were comparable in all seven of the studied strata and encompassed animals that were similar with respect to sex, age and seropositivity. The proportion of dogs presenting positive Dual Path Platform (DPP®) immunoassay tests was 10.8% (76/704), whereas the global prevalence of seropositive dogs, i.e. those that were both DPP-reactive and positive in the confirmatory enzyme-linked immunosorbent assay (ELISA), was 6.7% (47/704). The medium ages of seropositive dogs (5 years) and their seronegative counterparts (4 years) were not significantly different according to the Mann–Whitney test (*p* = 0.234).

All variables related to better dog care were associated with lower chances of CVL. Bivariate analysis (^χ2^ test) revealed that the prevalence of CVL was associated significantly with guardians lacking formal education and with dogs adopted as adults, sheltered outside the domicile, allowed free access to the streets and fed with alternative types of food rather than commercial chow (Table 1). The final multiple regression model (MRM) (Table 2) showed that, considering primary or secondary education as a reference category, the chances of an animal developing CVL were significantly higher when their guardian had no formal education or possessed a university degree. In addition, there was a significantly greater chance of CVL in dogs raised exclusively in the peridomicile and with free access to the street in comparison with those raised within the confines of the domicile. In addition, dogs that were acquired as puppies presented half of the chance of developing the disease in comparison with those adopted as adults.

**Table 1:** Bivariate analysis showing the relationship between predictors of CVL and the results of serology tests

Predictor variable	Serology tests				*P* value
	**Seropositive**^a^ ***n*(%)**		**Seronegative *n*(%)**		
**Dog traits**					
Sex					
Female	25 (6.5)		360 (93.5)		0.831
Male	22 (6.9)		297 (93.1)		
Size					
Small	22 (5.4)		386 (94.6)		0.328
Medium	17 (7.9)		198 (92.1)		
Large	7 (8.9)		72 (91.1)		
Breed					
Purebred	17 (5.7)		281 (94.3)		0.421
Mixed-breed	29 (7.2)		372 (92.8)		
Hair length					
Short	37 (7.2)		475 (92.8)		0.242
Long	9 (4.8)		180 (95.2)		
Time in the domicile					
< 1 year	6 (5.3)		107 (94.7)		0.551
> 1 year	37 (6.8)		504 (93.2)		
**Socioeconomical status of guardian**					
Education					
No formal education		6 (20)		24 (80)	0.008
Incomplete primary school		16 (6.8)		221 (93.2)	
Complete primary or secondary school		17 (4.8)		336 (95.2)	
University degree		8 (9.8)		74 (90.2)	
Number of bathrooms in the domicile					
1	21 (5.9)		333 (94.1)		0.471
> 1	25 (7.3)		318 (92.7)		
**Ecological features of the domicile**					
Close to forest					
Yes	42 (7.4)		527 (92,6)		0.142
No	5 (3.8)		126 (96.2)		
Presence of chickens and other birds					
Yes	22 (8.3)		243 (91.7)		0.164
No	24 (5.6)		405 (94.4)		
Presence of cats					
Yes	14 (9.5)		133 (90.5)		0.120
No	33 (5.9)		524 (94.1)		
**Responsible animal guardianship**					
Dog age at the time of acquisition					
Adult	17 (10.9)		139 (89.1)		0.015
Puppy	29 (5.4)		508 (94.6)		
Mode of acquisition of dog					
Purchase	4 (3.8)		101 (96.2)		0.451
Born in the domicile	5 (6.5)		72 (93.5)		
Adopted	37 (7.2)		480 (92.8)		
Number of dogs in the domicile					
1	15 (6.7)		209 (93.3)		0.933
> 1	31 (6.5)		444 (93.5)		
Shelter conditions					
Exclusively in the domicile	1 (1.0)		95 (99.0)		0.034
Exclusively in the peridomicile	32 (8.3)		355 (91.7)		
In the domicile and peridomicile	13 (5.9)		207 (94.1)		
Access to the streets					
Yes	8 (14.8)		46 (85.2)		0.013
No	39 (6)		611 (94)		
Vaccination against rabies (at least once)					
Yes	43 (6.7)		603 (93.3)		0.411
No	2 (3.8)		51 (96.2)		
Worm treatment (at least once)					
Yes	38 (6.2)		573 (93.8)		0.190
No	8 (10.1)		71 (89.9)		
Tick infestation					
Yes	15 (8.3)		166 (91.7)		0.533
No	32 (6.2)		487 (93.8)		
Flee infestation					
Yes	14 (7)		187 (93)		0.796
No	33 (6.6)		464 (93.4)		
Fed with commercial chow					
Yes	43 (6.3)		642 (93.7)		0.011
No	4 (21.1)		15 (78.9)		
Sterilization status (nutered/spayed)					
Yes	4 (4.7)		81 (95.3)		0.451
No	42 (6.9)		569 (3.1)		

^a^Dogs presenting positive results for Dual-path Platform test (screening) and enzyme-linked immunosorbent assay (confirmation)

**Table 2:** Final multiple regression model comprising the main predictors of CVL

Predictor variable	Odds ratio	*P* value	95% Confidence interval	
			Minimum	Maximum
Guardian with no formal education^a^	4.091	0.013	1.139	12.501
Guardian with incomplete primary school^a^	1.484	0.284	0.720	3.058
Guardian with university degree^a^	2.731	0.030	1.101	6.773
Dogs sheltered outside the house	2.033	0.037	1.042	3.964
Dogs with free access to the streets	2.471	0.040	1.043	5.854
Dogs acquired at the puppy stage	0.491	0.035	0.253	0.950

^a^Reference category comprised guardians with complete primary or secondary school education

**Fig. 1:**
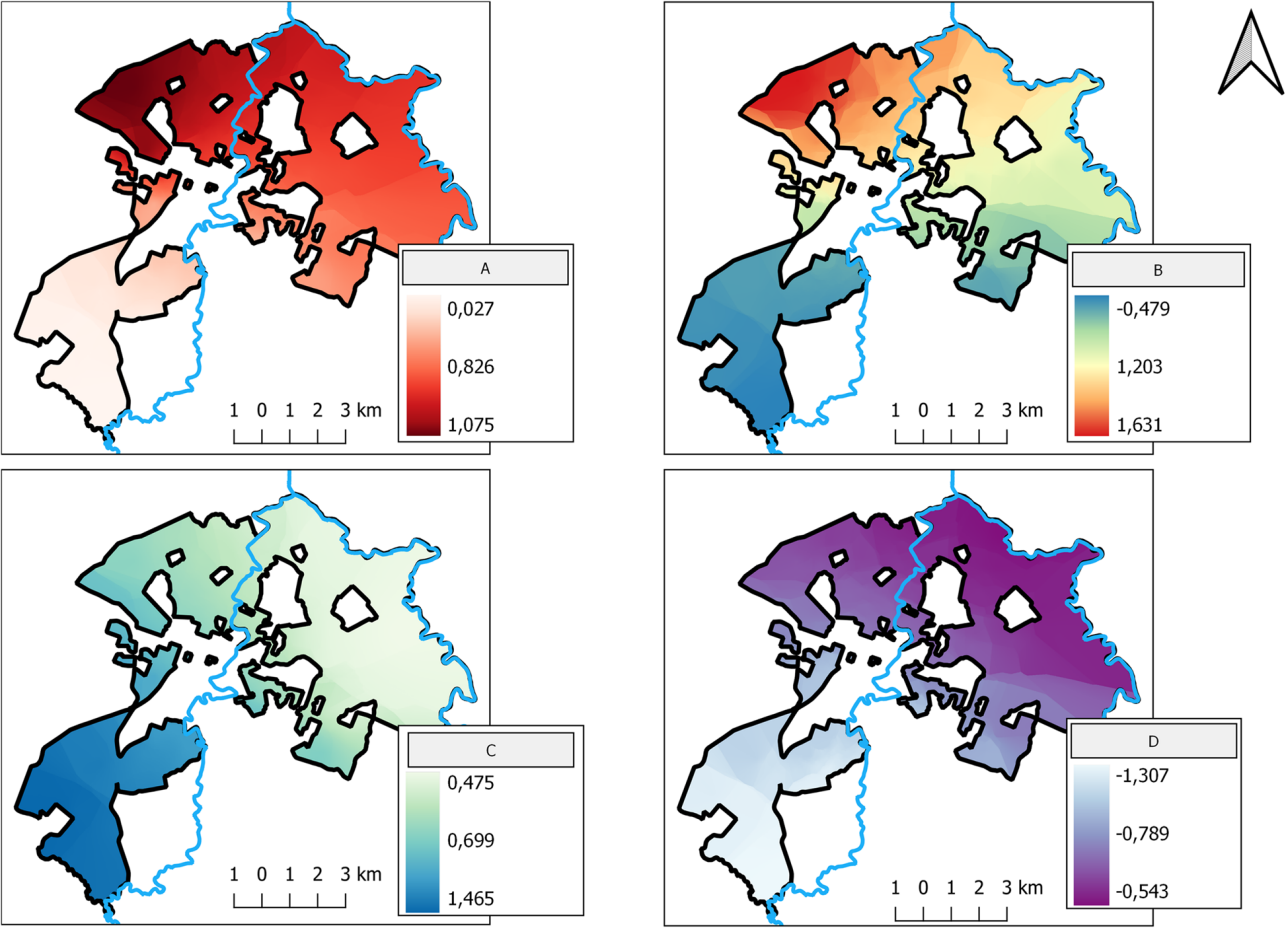
Geographically weighted logistic regression (GWLR) model of canine visceral leishmaniasis showing the predictor variables: **A**) Dogs acquired at the puppy stage; **B**) Guardians with no formal education; **C**) Dogs with free access to the streets, and **D**) Dogs sheltered outside the house. The rivers Itapecerica and Para that cross the city of Divinópolis are shown in blue; colors represent the strength of the associations within the study area as indicated in the respective inserts; positive values of the coefficients indicate a greater chance of CVL in the dogs of the indicated group

The final MRM exhibited a better fit than the geographically weighted logistic regression (GWLR) model, as confirmed by the lower bias-corrected Akaike information criteria (AIC_c_) score of the former (337.0 and 341.91, respectively). Nevertheless, data from the GWLR maps could be employed to observe the strengths of contributions that the predictor variables exerted on the occurrence of CVL over geographical space. As shown by the spatially distributed coefficients presented in Fig. 1, the associations in the MRM and GWLR models were always in the same directions but the strengths varied according to area.

## Discussion

In the present study, factors associated with the occurrence of CVL were analyzed in seven strata in the municipality of Divinópolis, and included dog-, guardian- and environment-related variables together with those of RCAG.

Our survey showed that dogs raised in the peridomicile and those with free access to the streets were at higher chance of developing CVL, as identified in previous studies [8–10]. The greater prevalence of the disease in such animals can be explained by the higher degree of exposure to the vectors of *Leishmania*. On this basis, public health strategies aimed at decreasing contact with sand flies, perhaps by the use of repellent collars, should be directed primarily to these animals [11]. The problems generated by unconfined dogs are more serious because of the important role played by these animals in the dissemination of CVL [12, 13]. In this context, surveillance of areas commonly used by free-roaming dogs, and education of guardians concerning their obligations and responsibilities towards the welfare of companion animals, are essential for the control of CVL and other zoonotic diseases [2].

A novel risk factor of CVL revealed by our study was the acquisition of dogs in the adult phase rather than as puppies. This result may be explained by the impracticality of a guardian to obtain complete information about the medical history of an older animal, particularly when adopting a stray dog. Such animals may have come from areas with high prevalence of CVL and/or may not have received proper health care, thereby increasing the risk of introduction/transmission of the disease in less affected areas. Our results suggest that, when adopting an adult dog, the animal should be subjected to a full clinical evaluation, including tests for CVL, regardless of age or mode of acquisition (adoption or purchase). Responsible acquisition represents an important RCAG principle that ought to be embraced by the public in order to prevent the spread of disease and the disorderly growth of the canine population [14].

The educational level of an animal’s guardian has not been fully explored in studies relating to CVL infection [15, 16]. The results presented herein revealed that the chances of a dog being seropositive for CVL are four-fold higher when the guardian has no formal education in comparison with those declaring complete primary or secondary schooling. Interestingly, dogs under the care of guardians with university degrees also had a greater chance of being seropositive, indicating that, although CVL is associated with worse socioeconomic conditions, it is not necessarily exclusive to poor areas. Another socioeconomic-related variable (number of bathrooms in the domicile) showed no association with CVL and, furthermore, the disease profile was similar in all analyzed strata.

Although many RCAG variables (mode of acquisition, number of dogs in the domicile, rabies vaccination, worm treatment, tick/flee infestation and sterilization status) were not associated significantly with CVL, the disease showed a higher prevalence in dogs that had not received adequate care, i.e. when RCAG principles were not entirely followed. Noting that vaccination against CVL and the use of repellent collars, along with other preventive measures, had not been adopted by guardians in the sampled domiciles, the isolated effects of the majority of the studied variables on the outcome were weak and not statistically significant. On the other hand, the directions of the associations showed that all of the predictor variables influenced the transmission of the zoonosis and, when considered together, could modulate the outcome and reduce the chances of infection. As is the case for many zoonotic diseases, CVL is driven by numerous external factors including non-compliance with RCAG, and any failure in applying the principles embodied therein jeopardizes public health and animal welfare [2]. Several authors have highlighted the need for governmental authorities to invest in education about RCAG [17–19], and our results reinforce the view that such measures should be addressed to both current and prospective guardians [20].

Other studied variables relating to dog traits and ecological features of the domicile showed relationships in directions consistent with those identified previously [7] but were not associated significantly with CVL in the present survey. The low strengths of those associations that have already been described in the literature emphasize the difficulty in understanding the disease and in establishing control actions based on the characteristics of the dogs.

The results of GWLR converged with those of the final MRM and allowed the identification of predictor variables that showed the strongest associations with CVL within the various areas of the municipality [21]. This type of analysis can be useful for directing municipal control actions, and its application should be encouraged in areas where CVL is a relevant public health issue.

Our study revealed that the global prevalence of CVL in the sampled dog population in Divinópolis was 6.7%, a value that differed from the prevalence of 13.6% reported by Penaforte et al. [22] and of 4.63% registered by Teixeira-Neto et al. [23]. Since our sampling method was analogous to that used in the study by Teixeira-Neto and coworkers [23], it is possible to infer that the prevalence of CVL in the urban area has not diminished, suggesting that the control measures adopted during the period between the two studies may have been relatively ineffective. Morais et al. [6] emphasized the importance of a long-term surveillance and control plan for CVL comprising defined goals, together with continuous monitoring and evaluation of the effectiveness of the activities by the public health services. One of the main issues in Divinópolis is the absence of serological surveys since 2014, a situation that contravenes the recommendations of the Brazilian Ministry of Health [24]. Even in the survey reported herein, it was not possible to obtain coverage of the entire municipality within one year following performance of the first test.

The study was subject to some limitations, one of which was that the survey only covered part of the urban area. However, the random sampling design, the adequate size and the diversity of the sample population conferred sufficient variability for the analysis of associations between the study predictors and CVL and for external validation of the data. The second limitation was the high percentage of domiciles that could not be found and of residents who were absent at the time of the survey. However, such sample losses were homogeneous across the studied area and, therefore, did not influence the results. The third limitation is that possible differences in communication skills of the interviewers could have produced varying responses, despite the standardized structure of the questionnaire and the consistent mode of the interviewing process that had been carefully planned by the researchers to prevent bias.

## Conclusions

Three RCAG predictors were associated significantly with CVL, namely dogs sheltered outside the house, dogs with free access to the streets and dogs acquired at the adult stage rather than as puppies. While the former two predictors have been identified in previous studies, the finding that the acquisition of puppies afforded a lesser chance of acquiring *Leishmania* infection is novel. Our results highlight the need to provide health education and to improve awareness and accountability of the population regarding the care, welfare, treatment and quality of life of their companion animals, considering that these actions could be positive effectors in the control of VL. Finally, the results have external validity and are applicable to similar Brazilian settings, in which inadequate dog care is a relevant public health problem.

## Methods

### Site of study and design

**Fig. 2:**
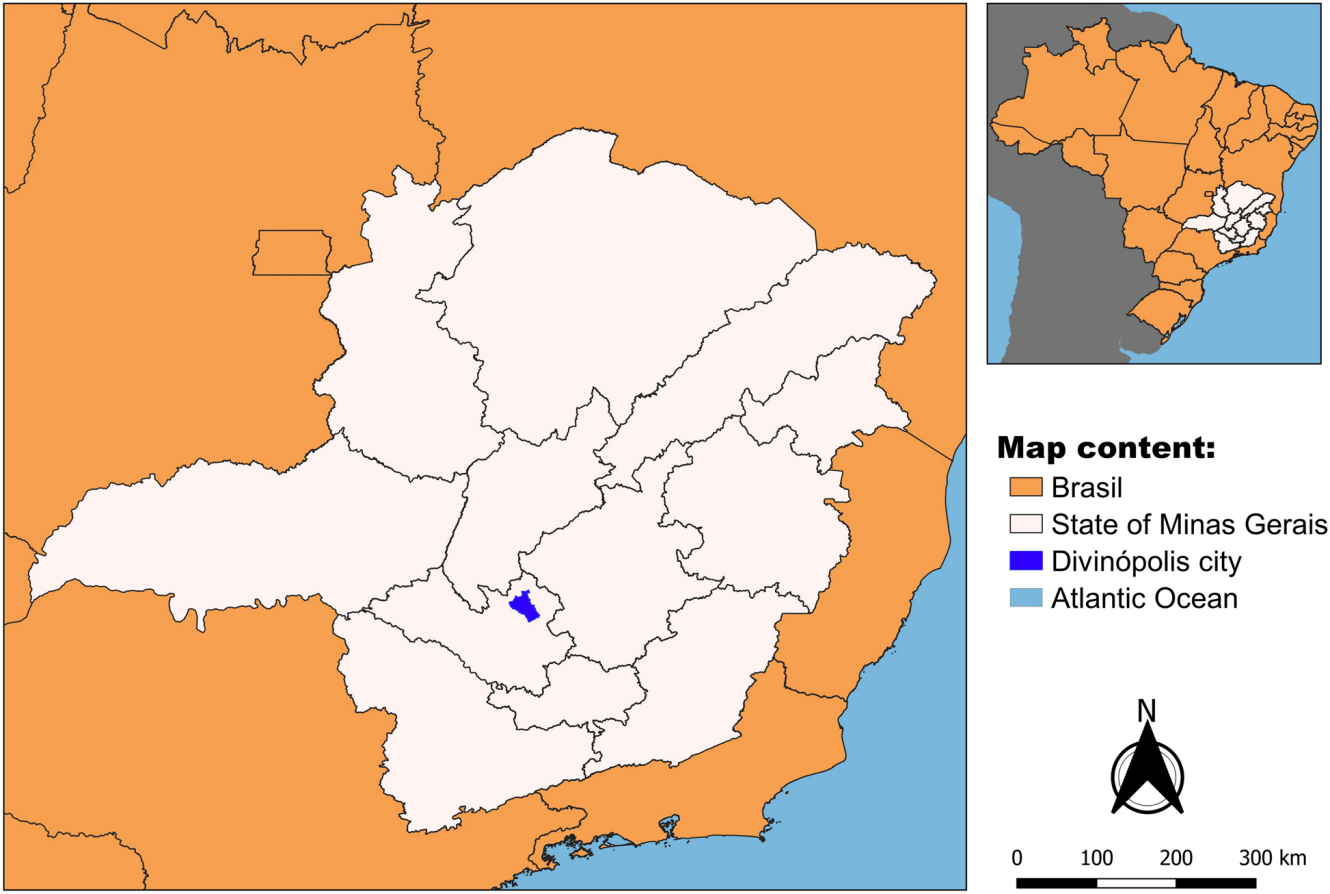
Study setting: Location of Divinópolis, Minas Gerais, Brazil

This analytical cross-sectional epidemiological study was carried out in Divinópolis, MG, Brazil, a city of approximately 240 thousand inhabitants [25] located in the metallurgical zone of the Itapecerica Valley micro-region and Upper São Francisco macro-region (Fig. 2). Between 2007 and 2019, twenty-five new confirmed cases of Human Visceral Leishmaniasis were reported in the municipality [26]. For the purposes of this study, the municipality of Divinópolis was divided spatially into 11 strata, as defined in the Plano Nacional de Controle da Dengue (PNCD) [27], in accordance with the procedure recommended by the Brazilian Ministry of Health for the control of VL. Each stratum was subdivided into blocks and districts or, in cases where such division was not covered by PNCD, blocks were created on the basis of the characteristics of the area, considering a delimitation by streets and size of 10,000 to 15,000 m2 [23].

The study was conducted in partnership with the local municipal government through the offices of SEMUSA and was carried out between January 2018 and May 2019. In the screening phase, technical staff from SEMUSA visited a number of randomly selected domiciles located within seven of the eleven strata of the urban area [23] in order to apply diagnostic tests for CVL. In the survey phase, the selected domiciles were visited by UFSJ researchers with the aim of collecting data regarding the predictor variables of CVL through application of a questionnaire.

The sample size of the study was defined on the assumption that each stratum encompassed a human population of approximately 17,000 residents, the proportion of dogs in the municipality was homogeneous, and the prevalence values of CVL in the strata were those reported by Teixeira-Neto et al. [23]. Larger numbers of diagnostic tests were performed in strata with lower prevalence values in order to maintain the accuracy of the estimates. The final sample size obtained in the study (704 dogs) made it possible to estimate a prevalence of 10%, with a confidence level of 95% and a margin of error of 2.2% in the estimate.

### Diagnosis of CVL

Initially, all dogs in a selected domicile were screened using DPP tests (Biomanguinhos, Rio de Janeiro, RJ, Brazil), following which blood samples were collected from all DPP- reactive dogs in order to confirm the diagnoses through ELISA tests performed at the Immunology and Parasitology Laboratory at UFSJ. Dogs presenting positive results in both DPP and ELISA were considered seropositive. The data obtained (i.e. addresses of the domiciles visited, numbers of dogs sampled and test results) were recorded in a Microsoft Excel® spreadsheet by staff at SEMUSA and subsequently transmitted to the UFSJ researchers involved in the study.

### Surveying process

The selected domiciles were subsequently visited by UFSJ researchers in order to apply a structured questionnaire about the predictor variables of CVL, namely: (i) dog traits—age, sex, breed, size, hair length, time in the domicile; (ii) socioeconomic status of the guardian—education of the head of the family, number of bathrooms in the domicile; (iii) ecological features of the domicile—proximity to forest areas (assessed by questioning residents about the existence of backyards with trees in the household; by observing *in loco* the presence of vegetated areas adjacent to the house and by viewing satellite images of the studied region), coexistence with cats, chickens and other birds; (iv) RCAG—dog age at the time of acquisition, mode of acquisition, number of dogs in the domicile, shelter conditions, access to the streets, vaccination against rabies, worm treatment, tick and flee infestation, food type, sterilization. A preliminary version of the questionnaire was drafted by researchers at UFSJ and a pilot test was applied to a subset of residents domiciled in an area not selected for the study. Based on the answers given by the respondents, adaptations were made to the questionnaire to improve the clarity of some aspects and to optimize the application time.

### Statistical analysis

Data collected from the questionnaire were entered into Epi Info™ (Centers for Disease Control and Prevention, Atlanta, GA, USA) software and analyzed using R (R Development Core Team, Vienna, Austria) or QGIS (QGIS Development Team; http://www.qgis.org) programs. The predictor variables were characterized by descriptive statistics, while the ages of seropositive and seronegative dogs were compared using the Mann–Whitney test. Bivariate analysis was employed to determine which of the predictor variables were significantly (*P* < 0.05) associated with the outcome (CVL). Variables that achieved *p* values < 0.20 in the bivariate analysis were included in simple logistic regression models. Procedures involving backward stepwise variable selection were performed in order to obtain the final MRM comprising predictor variables that were associated significantly (*P* < 0.05) with the outcome. The degree of association between the outcome and the predictor variables were expressed by the odds ratio (OR), 95% confidence interval and *P* value.

GWLR models were used to explore the spatial variability of the data set and the strength of associations within different areas of the studied space [21]. Analyses were performed using GWR4.0 software (https://gwrtools.github.io/gwr4-downloads.html) with outcome and predictor variables as in the final MRM. Maps were produced to display the strength of relationships between the outcome and predictor variables according to geographic space. Comparison of goodness-of-fit measures of the different constructed models was performed on the basis of AIC_c_ score.

## Supplementary Information

**Additional file 1.**

## Data Availability

All data generated or analyzed during this study are included in this published article [and its supplementary information files].
